# From *Saccharomyces cerevisiae* to *Candida glabrata* in a few easy steps: important adaptations for an opportunistic pathogen

**DOI:** 10.1111/j.1574-6968.2010.02102.x

**Published:** 2011-01

**Authors:** Andreas Roetzer, Toni Gabaldón, Christoph Schüller

**Affiliations:** 1 Max F. Perutz Laboratories, Department of Biochemistry and Cell Biology, University of ViennaVienna, Austria; 2 Comparative Genomics Group, Bioinformatics and Genomics Programme, Centre for Genomic RegulationBarcelona, Spain

**Keywords:** fungal pathogen, stress response, apoptosis

## Abstract

The opportunistic human fungal pathogen *Candida glabrata* is closely related to *Saccharomyces cerevisiae*, yet it has evolved to survive within mammalian hosts. Which traits help *C. glabrata* to adapt to this different environment? Which specific responses are crucial for its survival in the host? The main differences seem to include an extended repertoire of adhesin genes, high drug resistance, an enhanced ability to sustain prolonged starvation and adaptations of the transcriptional wiring of key stress response genes. Here, we discuss the properties of *C. glabrata* with a focus on the differences to related fungi.

## Introduction

**Fig. 1:**
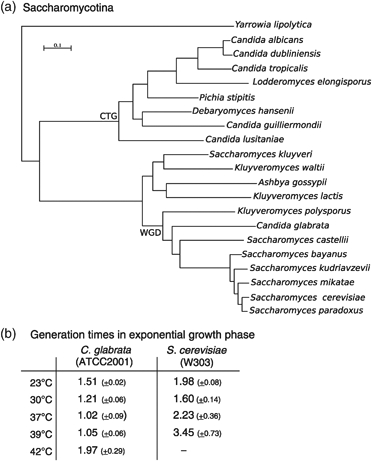
(a) Phylogenetic tree representing the evolutionary relationships between sequenced *Candida* and *Saccharomyces* species (adapted from T21 tree in Marcet-Houben & Gabaldón, 2009). The tree is based on the maximum likelihood analysis of concatenated alignments of 1137 protein families that have single-copy orthologues in the 21 species considered. Approximate likelihood ratio test support values were >0.95 for all branches, except for the one joining *Debaryomyces hansenii* and *Candida guilliermondii* (0.25). The genetic code transition in the CTG clade and the WGD events are indicated in the tree. (b) Generation time of *Candida glabrata* and *Saccharomyces cerevisiae* strains at different growth temperatures based on OD_600 nm_ measurements during exponential growth phase in rich medium (three experiments, SDs in parentheses).

Among the non-*Candida albicans Candida* species (Kaur *et al.*, 2005; Marcet-Houben & Gabaldón, 2009;), *Candida glabrata* is the species most closely related to *Saccharomyces cerevisiae* (Fig. 1a). In this review, we will discuss some of the properties that allow *C. glabrata* to exist as a human commensal. We will briefly discuss the infectivity, genetic changes, drug and stress resistance as well as the adherence of *C. glabrata*. A selection of traits supporting commensalism and/or pathogenicity was proposed earlier on for fungal and bacterial pathogens. This might occur in the environment (Bliska & Casadevall, 2009), in confrontation with microbial communities or immune responses (Seider *et al.*, 2010). A striking adaptation of *C. glabrata* might be its rapid growth and short generation time, which is tuned to 37 °C (Fig. 1b). *Candida glabrata* rarely penetrates tissue and is therefore exposed to microbial competitors on mucosal surfaces. Additionally, *C. glabrata* does not undergo a sexual cycle allowing the generation of resistant spores. We believe that these restrictions, combined with the host environment, favored the development of several specific traits, for example its high resistance to starvation. Compared with *S. cerevisiae, C. glabrata* shows only a small number of genetic adaptations. Considering its phylogenetic position (Fig. 1a), it can be assumed that *C. glabrata*'s ability to infect humans emerged independently from that of other *Candida* species.

After *C. albicans, C. glabrata* is the most common cause of vaginal and oral candidiasis. *Candida glabrata* is the second most important species in terms of candidiasis in the United States (Pfaller & Diekema, 2004). In total, *C. glabrata* accounts for roughly 15–20% of all *Candida* infections, with this relative incidence increasing every year. In healthy individuals, *C. glabrata* is restricted because of the action of the innate immune system and the microbial communities, which counteracts dissemination by competition for nutrients and secretion of toxins (Wargo & Hogan, 2006; Pamer, 2007;). Immunocompromised persons suffering from neutropenia (e.g. cancer or transplant patients) or persons treated with antibiotics are much more susceptible to *C. glabrata* infections (Perlroth *et al.*, 2007; Caston-Osorio *et al.*, 2008; Cohen *et al.*, 2010;). *Candida glabrata* is more often found in elderly patients (Hof, 2010). In murine infection models, *C. glabrata* infection is cleared and mice need to be rendered neutropenic to make them susceptible to pathogenic yeast infections (MacCallum *et al.*, 2006). Together, this might indicate that *C. glabrata* is less virulent than *C. albicans* and mainly causes disease in severely ill patients.

## Be creative: genetic changes

*Candida glabrata* shares a recent common ancestor with several *Saccharomyces* species, and clearly belongs to a clade different from that of other *Candida* species which display particular features such as the recoding of the CUG codon to Serine (Fig. 1a). As a result of this evolutionary relatedness, most *S. cerevisiae* genes have orthologues in *C. glabrata* and the chromosomal structure in terms of gene order is largely conserved between the two species (Dujon *et al.*, 2004; Marcet-Houben & Gabaldón, 2009;). Among many others, shared genomic features include a similar stress response (Roetzer *et al.*, 2008) and a respiratory metabolism characterized by the lack of Complex I (Marcet-Houben & Gabaldón, 2009). Despite this general resemblance, several differences in terms of gene content can be found, which might play an important role with respect to the phenotypic differences. Compared with *S. cerevisiae, C. glabrata* seems to have undergone an increased rate of gene loss from their common ancestor (Dujon *et al.*, 2004). Notable absences include genes of the galactose, phosphate, nitrogen and sulfur metabolism. Similar to many other microbial pathogens (Ehrlich *et al.*, 2008), such a reductive evolution could be related to *C. glabrata*'s adaptation as mammalian commensal and opportunistic pathogen. Indeed, *C. glabrata* relies on its host to overcome important auxotrophies such as those of nicotinic acid, pyridoxine and thiamine (Kaur *et al.*, 2005). Conversely, *C. glabrata* encodes genes that are absent from other *Saccharomyces*, including a putative racemase of bacterial origin (Marcet-Houben & Gabaldón, 2010). Other differences entail gene expansions that are specific to the *C. glabrata* lineage. Three of these specific expansions affect cell-wall organization and are probably related to adhesion properties of *C. glabrata* cells. These expansions include six copies of extracellular glycosylphosphatidylinositol-linked aspartyl proteases, eight copies of a α-1,3-mannosyltransferase involved in cell-wall biogenesis and a variable number of glycosylphosphatidylinositol-linked epithelial adhesin (*EPA*) genes located in subtelomeric regions. Importantly, this last family, which is required for epithelial cell adhesion and biofilm formation, seems to be a unique adaptation of *C. glabrata* because no significant homology can be detected in *S. cerevisiae*. Nevertheless, *FLO* genes in *S. cerevisiae* coding for glycosylphosphatidylinositol-linked flocculins are usually considered to be functional homologues of *C. glabrata EPA* genes (De Las Penas *et al.*, 2003). Similarly to *FLO* genes, *EPA* genes are located in subtelomeric regions and are subject to SIR-mediated silencing (Kaur *et al.*, 2007). Moreover, *FLO* genes have been found to be involved in cell adhesion and biofilm formation in *S. cerevisiae* (Reynolds & Fink, 2001). An intriguing possibility is that *FLO* and *EPA* genes are evolutionarily related but their sequences have diverged beyond recognition. Such an accelerated divergence seems to be quite frequent in yeast genes (Wolfe, 2004) and may have been favored by the extensive lineage-specific duplications undergone in the two lineages and by the acquisition of different tandemly repeated motifs.

Interestingly, despite being composed of different amino acids, the length of the tandemly repeated motifs in several *FLO* and *EPA* genes is similar (45 amino acids), perhaps reflecting a common selective pressure (Thierry *et al.*, 2010). Finally, a recently discovered feature of the *C. glabrata* genome, one that is almost unique to this species, is megasatellites (Thierry *et al.*, 2010). These long (135–417 nucleotides) repetitive sequences are enriched within genes coding for cell-wall proteins, including some *EPA* genes, and might also be related to the high degree of genomic plasticity observed in *C. glabrata* (Polakova *et al.*, 2009). This genomic plasticity is likely to play an important role in the evolution of *C. glabrata* populations because a sexual cycle has not yet been observed. Remarkably, this apparent disappearance of the sexual cycle seems to be a common trait for most fungal pathogens (Butler, 2010). Thus, differences in the genome sequence and its regulation provide important hints about the way in which two closely related yeasts have adapted to radically different environments.

## Be sticky: adherence and persistence

Compared with *S. cerevisiae, C. glabrata* has lost some of the genes needed for several metabolic pathways, for example galactose utilization. However, it has also gained certain functions necessary for its commensal lifestyle. Unlike *S. cerevisiae, C. glabrata* is able to adhere not only to mammalian cells but also to other surfaces (Cormack *et al.*, 1999). An elegant screen for factors required for biofilm formation identified *CgRIF1, CgSIR4* as well as the protein kinase *CgYAK1* and *CgEPA6* encoding an adhesin (Iraqui *et al.*, 2005). CgEpa6 is a member of the *EPA* gene family of glycosylphosphatidylinositol-anchored cell-wall proteins (Castano *et al.*, 2005; Domergue *et al.*, 2005; Iraqui *et al.*, 2005; Kaur *et al.*, 2005;). Similar to the *S. cerevisiae FLO* lectin-like genes, the *EPA* genes are encoded in subtelomeric clusters and are subject to transcriptional silencing (Castano *et al.*, 2005). *Candida glabrata* cells with mutations in the telomeric silencing factors Sir2, Sir3 and Sir4 are more adherent to cultured epithelial cells and show better colonization efficiency in kidneys (Castano *et al.*, 2005; Domergue *et al.*, 2005;). Interestingly, the lack of nicotinic acid, a precursor of NAD^+^, triggers adhesin (*EPA6*) expression (Domergue *et al.*, 2005). *Candida glabrata* is an auxotroph for NAD^+^ and utilizes nicotinamide riboside and nicotinic acid as NAD(+) sources during disseminated infection (Ma *et al.*, 2007). The majority of all genes transcriptionally induced by niacin limitation are also regulated by the NAD(+)-dependent histone deacetylase Hst1 (Ma *et al.*, 2009). Therefore, nicotinic acid and nicotinamide riboside are important host-derived regulatory signals. Transcript profiling of *C. glabrata* cells internalized by macrophages revealed increased expression of a gene family encoding extracellular glycosylphosphatidylinositol-linked aspartyl proteases (*YPS* genes) (Kaur *et al.*, 2007). YPS proteases are responsible for removing and releasing glycosylphosphatidylinositol-anchored cell-wall proteins and are thought to be necessary for cell-wall integrity and regulated adherence to mammalian cells. A recent survey identified several putative adhesin genes that had not yet been annotated (de Groot *et al.*, 2008). Their role for *C. glabrata* virulence is currently unknown. It should also be noted that *C. glabrata* strains have different number and sequence of adhesin genes. Thus, *C. glabrata* uses signals from the host for growth and adherence and its adhesive repertoire is more variable and by far transcends that of *S. cerevisiae*.

## Be adaptive: environmental stress response (ESR)

Vegetative cells of *C. glabrata* have high intrinsic stress tolerance. While *C. albicans* can survive on surfaces up to 4 months, *C. glabrata* can do so for even up to 5 months (Kramer *et al.*, 2006). However, the persistence traits have not yet been explored systemically. Stress resistance might be a consequence of its natural habitat. *Candida glabrata* preferably grows on mucosal surfaces as biofilm. Mammalian mucosal areas cause nutrient shortage, osmotic and other stresses due to the presence of other microorganisms and protective mechanisms of the host (Garside *et al.*, 2004; Pamer, 2007;). Furthermore, sexual reproduction as a way to generate stress-resistant spores has never been observed in *C. glabrata* (reviewed in Butler, 2010). So far, all clinical isolates of *C. glabrata* have been haploid, although it has the components of the mating machinery at its disposal (Wong *et al.*, 2003). *Candida glabrata* might have lost the sexual reproduction cycle relatively recently or perhaps suppresses it in order to maximize proliferation.

Changing their transcriptional program is a major strategy of microorganisms to adapt to their immediate environment. In *S. cerevisiae*, the ESR comprises about 900 genes whose expression is coordinately altered to exposure of different types of stress (Gasch *et al.*, 2000). *Saccharomyces cerevisiae, C. albicans, Schizosaccharomyces pombe* and *C. glabrata* live in different environments. Transcript analysis revealed that environmental stress (heat, hyperosmolarity, oxidative and starvation stress) induces a very similar pattern of regulated genes in these fungi (Chen *et al.*, 2003; Enjalbert *et al.*, 2003; Gasch, 2007;). Environmental stress activates a variety of conserved signaling mechanisms. Each of them responds to a particular cue, for example oxidative stress or carbon source starvation. Among them, mitogen-activated protein kinase (MAPK) pathways are central for relaying stress and other environmental signals. The ESR of *C. albicans* and *S. pombe* is mainly regulated by the stress-activated MAPKs CaHog1 and Sty1, respectively (Smith *et al.*, 2004; Enjalbert *et al.*, 2006;). CaHog1 (and most likely also *C. glabrata* CgHog1) is for full virulence (Alonso-Monge *et al.*, 1999). In *S. cerevisiae*, the high osmolarity glycerol (HOG) MAPK pathway senses osmotic stress and, to some extent, also other stress types such as oxidative stress and acetate (Smith *et al.*, 2010). The function of the *C. glabrata* HOG pathway seems to be very closely related to that of *S. cerevisiae*. However, unlike the *S. cerevisiae* HOG pathway, it also modulates resistance to longer chain weak organic acids such as sorbic acid (Gregori *et al.*, 2007). Sorbic acid is a powerful activator of the stress transcription factors Msn2 and Msn4 in *S. cerevisiae* but not of the orthologous factors in *C. glabrata* (Schüller *et al.*, 2004; Roetzer *et al.*, 2008;). Sorbic acid leads to rapid localization of CgMsn2-CFP to the nucleus in *S. cerevisiae* but not in *C. glabrata*. The difference suggests an as yet unknown weak acid signaling mechanism triggering the HOG pathway in *C. glabrata*. In *S. cerevisiae*, the activation of the HOG pathway is triggered by two redundant cell surface membrane sensors, Sho1 and Sln1. These genes have orthologues in *C. glabrata*. Initial genetic analysis of the first completely sequenced *C. glabrata* strain [CBS132, ATCC 2001 (Dujon *et al.*, 2004)] suggested that the Sln1 signaling branch was inactive because mutant strains lacking *CgSHO1* displayed a high sensitivity to osmotic stress (Calcagno *et al.*, 2005; Gregori *et al.*, 2007;). Further analysis revealed that a component of the Sln1 branch in this particular *C. glabrata* strain harbors a nonsense mutation (*Cgssk2*-*1*), leading to its inactivation. Importantly, other strains (e.g. BG2) do not share this allele.

In *S. cerevisiae*, the partially redundant transcription factors Msn2 and Msn4 regulate many ESR genes in parallel to MAPK pathways. Msn2 and Msn4 also convey nutrient signals and are regulated by protein kinase A (Görner *et al.*, 2002). Most Saccharomycotina species contain orthologues to *MSN2*. One important functional similarity of ScMsn2 orthologues and paralogues is a conserved stress-regulated nuclear export signal (NES) (Görner *et al.*, 1998; Roetzer *et al.*, 2008;). Msn2 orthologues from the CTG clade (Fig. 1a) do not have the stress-regulated NES and are most probably not involved in stress response. In the *C. albicans*, the Msn2-like protein (CaMsn4) lacks recognizable homology to the ScMsn2 NES and does not play any role in stress response (Nicholls *et al.*, 2004). Because of selection against genetic redundancy, most gene pairs that originated during the whole-genome duplication (WGD) event lost one of the copies in subsequent lineages. Thus, retention of both Msn2 and Msn4 in many species (with the exception of *Saccharomyces bajanus*) suggests different roles for these factors. Like other post-WGD species, *C. glabrata* encodes orthologues to both Msn2 and Msn4. CgMsn2/4 are important regulators for the CgESR but seem to be less relevant for glucose signaling (Roetzer *et al.*, 2008). So far, CgMsn2/4 have not been involved in pathogenicity and thus might play a role in persistence outside the host (Mundy & Cormack, 2009). These stress response factors might control the balance between stress resistance vs. growth required during feast and famine cycles (Berry & Gasch, 2008).

Protective response to oxidative stress is important for a pathogen facing oxidative burst attacks of innate immune cells. Compared with common *S. cerevisiae* laboratory strains, *C. glabrata* possesses high intrinsic oxidative stress resistance, which is regulated, similar to that of *S. cerevisiae*, by the transcription factors CgSkn7, CgYap1 and CgMsn2/4 (Cuellar-Cruz *et al.*, 2008; Roetzer *et al.*, 2010;). It also produces an indole-derived pigment contributing to oxidative stress resistance (Brunke *et al.*, 2010). The loss of CgMsn2/4, CgYap1 and CgSkn7 did not affect the survival of *C. glabrata* when confronted with murine macrophages. Interestingly, the expression of the *C. glabrata* superoxide dismutase genes (*CgSOD1*/*2*) is different from that of *S. cerevisiae. CgSOD1* seems to be expressed constitutively, and both *CgSOD1* and *2* are induced by glucose starvation. *Candida albicans* is equipped with five *SOD* genes with highly specialized roles during host contact (Frohner *et al.*, 2009). A slight adaptation of *SOD* regulation adapts *C. glabrata* to an environment with a higher risk of oxidative stress. In addition, many genes of the oxidative stress regulon can also be induced by other stresses such as glucose starvation (our unpublished data). Another adaptation of transcriptional regulation between *C. glabrata* and *S. cerevisiae* is the regulation of the catalase gene *CgCTA1*. The single catalase of *C. glabrata* combines both the different transcriptional regulation (carbon source vs. stress) and the different intracellular localization (cytoplasm vs. peroxisomes) of the two *S. cerevisiae* catalases (Roetzer *et al.*, 2010). Thus, *C. glabrata* achieves a similar regulated and localized catalase activity, albeit by the regulation of one gene.

It was recently reported that certain *Candida* species can suppress the production of reactive oxygen species (ROS) produced by phagocytic cells in murine and human phagocytes, whereas internalization of *S. cerevisiae* cells enhanced ROS production (Wellington *et al.*, 2009). Interestingly, in a cell-free system, *S. cerevisiae* was able to scavenge ROS in a manner similar to the one observed for *C. albicans*. Therefore, scavenging alone cannot account for the suppression of ROS production. We assume that an additional *Candida*-specific mechanism is responsible for active suppression. These results are supported by the finding that the transcription pattern of *C. glabrata* cells during phagocytosis did not show a prominent upregulation of oxidative stress-associated genes (Kaur *et al.*, 2007). This might be the result of the efficient suppression and/or detoxification of ROS due to expressed protective enzymes. The role of the stress response for *C. glabrata* adaptation to the host environment is still being studied. So far, the available evidence points to a range of subtle changes of mechanisms known from *S. cerevisiae*.

## Be flexible: phenotypic and morphological switching

Being largely immotile, fungi rely on passive dissemination methods and directed growth to expand into substrate. *Candida glabrata* primarily grows on surfaces and normally does not penetrate tissue. Under appropriate conditions, penetration into solid medium can be observed. For *C. albicans*, the switch between yeast and hyphae is one important contribution to virulence (Kumamoto & Vinces, 2005). In contrast, and similar to *S. cerevisiae, C. glabrata* can form pseudohyphae in response to nitrogen source starvation (Csank & Haynes, 2000). In *S. cerevisiae*, pseudohyphal growth is assumed to be a common event in search for nutrient-rich, optimal growth substrates. This morphological switch is regulated by a MAPK cascade, which has the transcription factor Ste12 as its final target (Gancedo, 2001). CgSte12 is also essential for the nitrogen starvation-induced formation of pseudohyphae in *C. glabrata*. In a mouse model, *Cgste12*Δ mutants show attenuated virulence (Calcagno *et al.*, 2003). However, pseudohyphal forms of *C. glabrata* have not yet been found in clinical specimens, possibly because this is a rare event (Kaur *et al.*, 2005).

Differently to pseudohyphal growth resulting from specific nutrient limitations, unusual cell aggregates have been observed for *C. glabrata* cells due to the loss of transcription factor CgAce2. These mutants have a cell separation defect and display a clumping phenotype that is similar to *S. cerevisiae ace2*Δ mutants (Kamran *et al.*, 2004). Interestingly, *Cgace2*Δ mutants are hypervirulent in a mouse model and are able to escape from the vasculature and penetrate into tissue (MacCallum *et al.*, 2006). Stead *et al.* (2010) found that lack of CgAce2 also changes the secretome of *C. glabrata*, which might contribute to its hypervirulence. The phenotype of the *Cgace2*Δ mutant supports the assumption that *C. glabrata* has a limited but possibly important ability to actively invade tissues despite its preferred growth on surfaces.

Phenotypic variability is important for evading the immune system. For *C. albicans*, switching between white and opaque cells is important for virulence (Kvaal *et al.*, 1997, 1999). White cells are more suited for bloodstream infections, whereas opaque cells colonize skin surfaces (Lachke *et al.*, 2003). *Candida glabrata* cells can undergo reversible phenotypic switching among phenotypes distinguishable by colony coloration on CuSO_4_-containing agar (white to very dark brown) and by the irregular wrinkle colony morphology (Lachke *et al.*, 2002; Brockert *et al.*, 2003;). In a mouse model, dark brown cells were reported to have an advantage in colonizing the spleen and the liver (Brockert *et al.*, 2003; Srikantha *et al.*, 2008;). The basis and molecular details of the reported phenotypic variability have not yet been explored in depth.

## Be resistant: drug resistance

One of the reasons for the success of *C. glabrata* is its relatively high drug resistance, in particular toward different azole antifungals. In the last decade, *C. glabrata* emerged as a cause of mucosal and invasive fungal infections, in part due to its intrinsic or easily acquired resistance to azole antifungals. Therefore, echinocandins such as caspofungin are the treatment of choice. The global transcriptional response to azole treatment and the molecular mechanisms to this drug resistance are reminiscent of *S. cerevisiae* (Vermitsky *et al.*, 2006). In *S. cerevisiae*, the zinc-cluster transcription factors ScPdr1 and ScPdr3 are central for regulating detoxification mechanisms based on transmembrane transporters such as ScPdr5 and ScSnq2. In *C. glabrata*, CgPdr1 activates the expression of *CgCDR1* and *CgCDR2*, which are drug efflux pumps of the ATP-binding cassette transporter type (Sanglard *et al.*, 2001). Gain of function mutations in *CgPDR1* enhances antifungal resistance and also increases virulence (Ferrari *et al.*, 2009). Additionally, defects in mitochondrial function causing respiratory deficiency also increase azole resistance and this, in turn, is linked to the upregulation of both *CgCDR1* and *CgCDR2* (Sanglard *et al.*, 2001). In both *S. cerevisiae* and *C. glabrata*, Pdr3 – and as a consequence drug resistance – is induced by the loss of the mitochondrial genome (S. Moye-Rowley, pers. commun.; Moye-Rowley, 2005). This might be a mechanism for a dedicated retrograde signal of drug-damaged mitochondria. Because the development of intrinsic drug resistance of *C. glabrata* preceded antifungal drugs, we speculate that these adaptations might be due to frequent exposure to toxic substances originating from the environment of the mucosal flora.

## Be aware of the source: nutrients

**Fig. 2:**
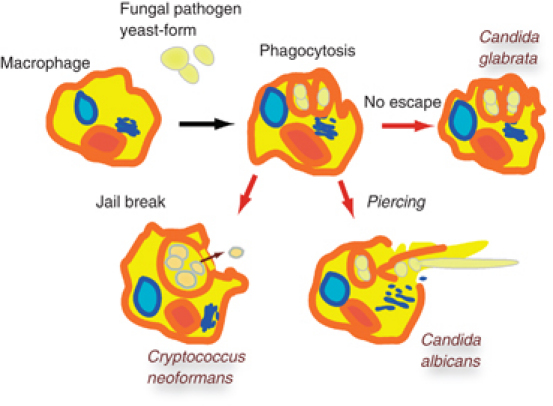
Fungal morphogenesis determines escape mechanisms of pathogenic yeasts. Upon engulfment by phagocytes, *Candida albicans* can form hyphae and escape from the phagosome by destroying the phagocyte. Instead of forming hyphae, *Cryptococcus neoformans* cells are able to escape by forming phagosomal extrusions. No specific mechanism to escape internalization is known for *Candida glabrata*.

Like other commensal or pathogenic microorganisms, *C. glabrata* faces phagocytic cells of the innate immune system during dissemination or after escaping from biofilms. Transcriptome studies revealed that *C. albicans, C. glabrata* and the encapsulated pathogenic yeast *Cryptococcus neoformans*, when engulfed by macrophages, switch their expression program to genes involved in the utilization of alternative carbon sources (Lorenz *et al.*, 2004; Fan *et al.*, 2005; Kaur *et al.*, 2007;). This is a default response, indicating loss of contact to the preferred carbon source glucose in the environment. Therefore, phagocytosed cells are sealed from the environment and have to rely on their endogenous resources or on those acquired from the phagocytic cell. Each successful pathogen has a unique strategy to manage this situation (Fig. 2). Unlike *C. albicans*, phagocytosed *C. glabrata* is unable to form hyphae and is trapped within the phagosome. *Cryptococcus neoformans* triggers phagosomal extrusions, which entails the escape of single cells without killing the phagocytes (Alvarez & Casadevall, 2006). The transcriptional response to phagocytosis also induced genes involved in autophagy, peroxisome function and lipid metabolism (Fan *et al.*, 2005). It is thought that carbon source and thus energy shortage is a predominant challenge for engulfed pathogenic fungi.

An important mechanism for survival during starvation conditions is autophagy (Klionsky, 2005). Autophagy is a continuous recycling process of cellular constituents and even organelles (Xie & Klionsky, 2007). Moreover, it is important for the virulence of several fungal pathogens of plants and animals (Hu *et al.*, 2008). In contrast, *C. albicans* escapes nutrient depletion by forming hyphae and killing engulfing macrophages. Consistently, mutants unable to induce autophagy survived challenge by macrophages (Palmer *et al.*, 2007). *Candida glabrata* trapped in a macrophage initially induced peroxisomes, most likely as part of the switch toward utilization of alternative carbon sources (Roetzer *et al.*, 2010). These peroxisomes were subsequently degraded via the pexophagy pathway, a specific subtype of autophagy. Both selective and bulk autophagy contribute to the survival of *C. glabrata* during engulfment. *Candida glabrata* trapped inside the phagosome requires mobilization of resources for survival. Because *C. glabrata* is relatively resistant to starvation, macrophages apparently have additional strategies to kill the internalized fungal cells. Macrophages are known to cause oxidative damage to microorganisms, but engulfed *C. glabrata* cells do not show signs of a severe oxidative stress load (Roetzer *et al.*, 2010). However, during maturation, the phagosome fuses with lysosomes in order to decrease the pH, which initiates enzymatic digestion of engulfed microorganisms (Stuart & Ezekowitz, 2005). *In vitro*, only the exposure to a combination of low pH and carbon starvation caused loss of viability of *C. glabrata*. The autophagy-deficient *atg11*Δ*atg17*Δ mutant was more sensitive to this particular combination of conditions, both *in vitro* and *in vivo*. Thus, the energy-demanding equilibration of extracellular pH with limited energy resources might be the challenging environment *C. glabrata* is exposed to in the phagosome.

## Conclusion

**Fig. 3:**
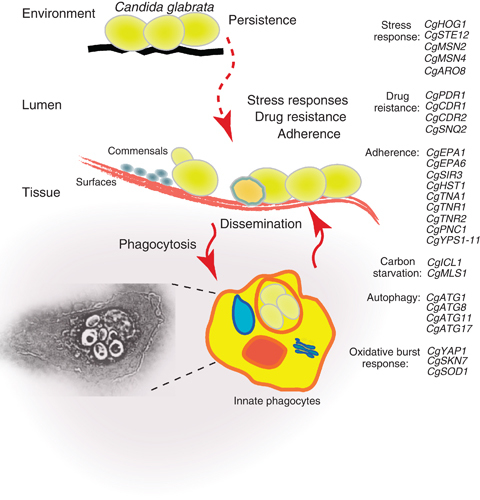
Possible habitats. *Candida glabrata* cells can persist on environmental surfaces for several months. As a commensal, *C. glabrata* is also found as part of the flora on interior mucosal areas of mammalians such as the gut. In immunocompromised persons, cells are able to disseminate into tissues and cause organ failure. Patrolling phagocytes are responsible for the extinction of invading yeast pathogens. The armory of *C. glabrata*: the right panel lists genes thought to be important for the opportunistic lifestyle of *C. glabrata*.

*Candida glabrata* is an efficient human pathogen, which can infect immunocompromised and elderly persons. Its avid adherence to mammalian tissue and to other surfaces is based on the expression of a number of specific adhesins. *Candida glabrata* rarely penetrates tissue actively and therefore directly competes with other microbial agents on mucosal surfaces. This might have led to the selection of specific drug and oxidative stress-resistance traits. Somatic fungal cells have high resistance to starvation, a trait that might also support survival during engulfment in phagocytic cells. Prolonged survival of *C. glabrata* inside phagocytic cells might support the establishment of disseminated infection and thus directly relate to its success as a commensal and pathogen (Fig. 3). Robust adherence, active suppression of fungicidal drugs, stress resistance and an enhanced ability to sustain prolonged starvation render *C. glabrata* superior to *S. cerevisiae* in causing disease. Altogether, we suggest that its adaptation to the mammalian host is rather due to slight genomic fine-tuning than due to radical large-scale changes.

## Statement

Re-use of this article is permitted in accordance with the Terms and Conditions set out at http://wileyonlinelibrary.com/onlineopen#OnlineOpen_Terms
